# Introducing Cardiac Magnetic Resonance in Athlete Screening: An Initial Moroccan Experience With Professional Football Players

**DOI:** 10.7759/cureus.102948

**Published:** 2026-02-04

**Authors:** Oumaima Taoussi, Youssef Daoudi, Zineb Azeddoug, Marwa Mokhtari, Hajar Rabii, Hibat Allah Kamri, Mohamed Amine Zahid, Soukaina Scadi, Ghali Benouna, Fatimazahra Merzouk

**Affiliations:** 1 Cardiology, Mohammed VI International University Hospital, Mohammed VI University of Health Sciences (UM6SS), Casablanca, MAR; 2 Cardiology, Cheikh Khalifa International University Hospital, Mohammed VI University of Health Sciences (UM6SS), Casablanca, MAR

**Keywords:** athlete’s heart, cardiac magnetic resonance, cardiomyopathy, echocardiography, pre-participation screening, sports cardiology, sudden cardiac death prevention

## Abstract

Background and objectives

Pre-participation cardiovascular screening in athletes relies primarily on electrocardiography and transthoracic echocardiography. While cardiac magnetic resonance (CMR) is increasingly recognized as a valuable complementary imaging modality, its integration into routine athlete evaluation remains variable across healthcare settings. In our context, the recent expansion of access to CMR has enabled its use as a second-line tool in professional athletes initially assessed by first-line screening examinations. This study aimed to describe CMR findings in asymptomatic professional football players referred after echocardiographic or electrocardiographic screening in a Moroccan tertiary center.

Methods

This descriptive study included 300 asymptomatic male professional football players aged 18 to 30 years who underwent routine pre-participation cardiovascular screening, including electrocardiography and transthoracic echocardiography, between January 2023 and June 2024. Among them, 40 players were referred for CMR based on clinical judgment following inconclusive echocardiographic findings or isolated electrocardiographic abnormalities. CMR findings were analyzed descriptively with respect to cardiac morphology, ventricular volumes, myocardial thickness, myocardial tissue characterization, and final diagnostic classification.

Results

Among the players referred for CMR, a broad spectrum of findings was observed. In several cases, CMR clarified borderline echocardiographic measurements by confirming physiological cardiac remodeling related to intensive football training. In other athletes, CMR identified previously unrecognized structural cardiomyopathies, including apical hypertrophic cardiomyopathy and a presentation suggestive of Fabry disease, which had not been detected on initial echocardiography. In players referred solely for isolated electrocardiographic abnormalities despite normal echocardiographic findings, CMR revealed clinically relevant myocardial abnormalities in a notable proportion of cases. Overall, CMR contributed to improved anatomical and tissue characterization in athletes with inconclusive first-line screening results.

Conclusion

In this descriptive Moroccan experience, CMR provided valuable morphological and tissue-level information in asymptomatic professional football players referred after initial cardiovascular screening. The growing accessibility of CMR in our setting has allowed more refined diagnostic evaluation beyond first-line examinations, particularly in athletes with equivocal findings. These observations highlight the practical role of CMR as a complementary imaging modality within contemporary athlete screening pathways in resource-evolving healthcare systems.

## Introduction

Sudden cardiac death in young competitive athletes, although rare, remains a devastating event with major medical, social, and media impact. Most underlying causes are structural or electrical cardiac diseases that may remain clinically silent until the first catastrophic presentation, making pre-participation cardiovascular screening a cornerstone of preventive sports cardiology [1,2]. Current screening strategies worldwide rely primarily on resting electrocardiography (ECG) and transthoracic echocardiography (TTE), which together allow detection of many cardiomyopathies, channelopathies, and congenital anomalies when appropriately interpreted [3].

However, physiological cardiac remodeling related to intensive athletic training-commonly referred to as the athlete’s heart-can overlap substantially with early or atypical forms of cardiomyopathy. Borderline left ventricular hypertrophy, equivocal wall thickness measurements, or subtle regional abnormalities may challenge conventional echocardiographic interpretation, particularly in high-level endurance and mixed-sport athletes such as professional football players [4,5]. Similarly, isolated ECG abnormalities may raise suspicion for underlying disease despite normal first-line imaging, creating diagnostic uncertainty and clinical dilemmas regarding eligibility and risk stratification [6].

Cardiac magnetic resonance (CMR) has emerged as the reference standard for the non-invasive assessment of cardiac volumes, myocardial mass, ventricular morphology, and tissue characterization. Its superior spatial resolution and reproducibility, combined with its ability to detect myocardial fibrosis, infiltration, or scarring using late gadolinium enhancement (LGE) and parametric mapping techniques, provide incremental diagnostic value over echocardiography [7,8]. CMR is particularly useful in differentiating physiological athletic remodeling from pathological entities such as hypertrophic cardiomyopathy, arrhythmogenic cardiomyopathy, and infiltrative myocardial diseases [9].

As a result, contemporary sports cardiology and imaging guidelines increasingly recognize CMR as a valuable second-line investigation in athletes with inconclusive or discordant findings on initial screening [3,10]. Nevertheless, the integration of CMR into routine athlete evaluation remains heterogeneous across healthcare systems and is strongly influenced by resource availability, cost, and local expertise. In many low- and middle-income countries, including Morocco, access to CMR has historically been limited, restricting its use to selected clinical indications.

In recent years, the expansion of CMR availability in Moroccan tertiary centers has progressively enabled its use in more nuanced clinical scenarios, including the evaluation of asymptomatic professional athletes referred after equivocal ECG or echocardiographic findings. However, real-world data describing the spectrum of CMR findings and its practical diagnostic contribution in athlete screening from resource-evolving healthcare systems remain scarce. The present study aimed to describe CMR findings in asymptomatic professional football players referred after initial cardiovascular screening in a Moroccan tertiary center, highlighting the added value of CMR as a complementary imaging modality in contemporary sports cardiology practice.

## Materials and methods

Study design and population

This was a descriptive, observational study conducted at a Moroccan tertiary referral center with dedicated expertise in cardiovascular imaging and sports cardiology. The study population consisted of asymptomatic male professional football players who underwent routine pre-participation cardiovascular screening between January 2023 and June 2024. All athletes were actively competing at a professional level and were referred through organized sports medical programs.

A total of 300 players aged 18 to 30 years underwent first-line cardiovascular evaluation including resting ECG and TTE. Among this cohort, 40 athletes were referred for CMR imaging following the initial screening assessment.

Indications for CMR

Referral for CMR was determined within a structured clinical framework involving cardiologists experienced in sports cardiology and cardiovascular imaging. Indications included equivocal or borderline echocardiographic findings, such as indeterminate ventricular wall thickness, borderline chamber dimensions, or limited acoustic windows, as well as isolated ECG abnormalities raising suspicion of underlying structural heart disease despite otherwise normal echocardiographic evaluation. Equivocal echocardiographic findings were defined as measurements or morphological features falling at the upper limits of normal for trained athletes or insufficient to confidently distinguish physiological athletic remodeling from pathological cardiomyopathy.

All equivocal ECG and echocardiographic findings were reviewed by at least two cardiologists with expertise in sports cardiology and cardiovascular imaging. Referral for CMR was based on a consensus decision and guided by contemporary international recommendations for second-line imaging in athletes with inconclusive first-line screening, applied pragmatically within the local healthcare context. All referred athletes were asymptomatic and had no previously known cardiovascular disease at the time of assessment.

ECG and echocardiographic assessment

Resting 12-lead ECGs were performed according to standard protocols and interpreted according to the International Criteria for Electrocardiographic Interpretation in Athletes. TTE was conducted by experienced operators using commercially available ultrasound systems. Standard parasternal and apical views were systematically acquired, allowing assessment of left and right ventricular dimensions, wall thickness, systolic function, and valvular morphology. When echocardiographic findings were considered inconclusive or discordant with ECG abnormalities, referral for CMR was considered.

CMR acquisition protocol

CMR examinations were performed using a 1.5-Tesla scanner equipped with a dedicated multi-channel cardiac coil. A standardized CMR protocol was systematically applied to all referred athletes, with minor adaptations performed when clinically indicated.

Cine imaging using breath-hold steady-state free precession sequences was acquired in standard long-axis views, including four-chamber, two-chamber, and three-chamber orientations, as well as in contiguous short-axis slices covering the entire left ventricle from base to apex. Dedicated right ventricular cine acquisitions were systematically performed, including focused right ventricular views and right ventricular outflow tract imaging, to allow comprehensive assessment of right ventricular morphology and function.

Myocardial tissue characterization included native T1 mapping performed prior to contrast administration, as well as T2-weighted short tau inversion recovery sequences to assess myocardial edema. Following intravenous administration of a gadolinium-based contrast agent, LGE imaging was acquired approximately 10 minutes post-injection using inversion-recovery sequences in standard long-axis views (four-chamber, two-chamber, and three-chamber) and matching short-axis slices. Additional right ventricular-focused LGE views were obtained when appropriate, and post-contrast T1 mapping was performed to further characterize myocardial tissue properties.

Ventricular volumes, ejection fractions, myocardial mass, wall thickness, and chamber dimensions were quantified using standard post-processing techniques in accordance with current CMR recommendations. All measurements and image analyses were performed by experienced readers with expertise in cardiovascular magnetic resonance.

Given the real-world, clinical nature of the study, CMR readers were aware of the referral indication; however, all measurements and diagnostic interpretations were performed according to standardized CMR criteria. All CMR examinations were acquired and interpreted by the same experienced cardiovascular magnetic resonance physician to ensure internal consistency of measurements and diagnostic interpretation.

Data collection and analysis

Clinical data, ECG findings, echocardiographic results, and CMR findings were retrospectively collected from medical records. CMR findings were analyzed descriptively with particular attention to cardiac morphology, ventricular volumes, myocardial thickness, and tissue characterization. Final diagnostic impressions were classified according to whether CMR findings were consistent with physiological athletic remodeling or suggestive of structural cardiomyopathy or myocardial disease.

Two cases identified within the study cohort, corresponding to apical hypertrophic cardiomyopathy and a Fabry disease-like myocardial phenotype, were previously reported separately as individual case reports. Genetic or enzymatic confirmation was not available at the time of screening. These cases were included in the present descriptive analysis solely to reflect the overall diagnostic spectrum observed during the study period and were not analyzed for novelty or duplication of findings. Given the descriptive nature of the study, no inferential statistical analyses were performed.

Ethical considerations

The study was conducted in accordance with the Declaration of Helsinki. Due to its retrospective and observational design, the requirement for written informed consent was waived according to institutional policy. All data were anonymized prior to analysis to ensure patient confidentiality.

## Results

Cohort characteristics and indications for CMR

Between January 2023 and June 2024, a total of 300 asymptomatic male professional football players (median age 23 years, range 18-30) underwent routine pre-participation cardiovascular screening, including resting ECG and TTE. Among this cohort, 40 athletes (13.3%) were referred for CMR imaging following the initial screening evaluation. Referral for CMR was performed within a selective institutional framework, reflecting the recent introduction of CMR into local screening protocols, limited availability of scanners, and restricted access to trained cardiovascular imaging specialists. All referred athletes were asymptomatic and had no previously known cardiovascular disease at the time of assessment.

Baseline characteristics of the screened population and the subgroup referred for CMR are summarized in Table 1, while the detailed echocardiographic findings prompting CMR referral are presented in Table 2. The primary indications for CMR referral were equivocal or borderline echocardiographic findings in 25 athletes (62.5%) and isolated ECG abnormalities despite a normal echocardiogram in 15 athletes (37.5%).

**Table 1:** Baseline characteristics of the screened population and athletes referred for cardiac magnetic resonance (CMR) ECG: electrocardiography

Characteristic	Total screening cohort (n = 300)	CMR subgroup (n = 40)
Age, years (median, range)	23 (18–30)	24 (18–30)
Male sex, n (%)	300 (100%)	40 (100%)
Asymptomatic at evaluation, n (%)	300 (100%)	40 (100%)
Referred for equivocal echocardiography, n (%)	–	25 (62.5%)
Referred for isolated ECG abnormalities, n (%)	–	15 (37.5%)

**Table 2:** Detailed breakdown of equivocal or borderline echocardiographic findings prompting CMR referral Note: some athletes presented more than one equivocal echocardiographic feature. LV: left ventricle; RV: right ventricle; ARVC: arrhythmogenic right ventricular cardiomyopathy; LVH: left ventricular hypertrophy; CMR: cardiac magnetic resonance

Category	Specific echocardiographic finding	Number of athletes (n = 25)
LV findings	Borderline maximal LV wall thickness (12–13 mm), indeterminate for LVH	15
	Borderline LV cavity dimensions	3
Right ventricular findings	Borderline RV dilatation and/or mildly reduced systolic function	5
	Of which: suspected ARVC phenotype on echocardiography	2
Technical limitations	Suboptimal acoustic windows limiting reliable ventricular assessment	2
Other ambiguous findings	Borderline chamber dimensions or non-specific structural abnormalities	3

Detailed description of equivocal or borderline echocardiographic findings

Among the 25 athletes referred for CMR due to equivocal echocardiographic findings, several recurrent patterns of diagnostic uncertainty were identified. The most frequent indication was indeterminate left ventricular hypertrophy, characterized by borderline maximal wall thickness values at the upper limits of normal for athletes or morphologic patterns insufficient to confidently differentiate physiological remodeling from early cardiomyopathy.

Right ventricular abnormalities constituted another important source of uncertainty, including borderline right ventricular dilatation or mildly reduced systolic function. In two athletes, these findings raised suspicion for arrhythmogenic right ventricular cardiomyopathy on echocardiography. Additional referral reasons included suboptimal acoustic windows limiting reliable assessment of ventricular morphology, as well as borderline chamber dimensions or other ambiguous structural findings. A detailed breakdown of these echocardiographic findings is provided in Table 2.

Spectrum of CMR findings and diagnostic reclassification

CMR imaging revealed a broad spectrum of findings among the referred athletes. In 32 athletes (80%), CMR confirmed physiological cardiac remodeling consistent with athletic adaptation. This group was characterized by symmetrical biventricular enlargement, preserved systolic function, increased ventricular volumes and myocardial mass exceeding sedentary reference ranges, and complete absence of LGE or abnormal parametric mapping findings.

Notably, this group included two athletes in whom arrhythmogenic right ventricular cardiomyopathy had been suspected on echocardiography. In both cases, CMR ruled out this diagnosis by demonstrating normal right ventricular volumes and systolic function, absence of regional wall motion abnormalities, and no evidence of right ventricular or left ventricular LGE. No athlete fulfilled CMR diagnostic criteria for arrhythmogenic cardiomyopathy.

In contrast, CMR established a definitive diagnosis of structural cardiomyopathy in five athletes (12.5%). These included one case of apical hypertrophic cardiomyopathy with marked apical hypertrophy (maximal wall thickness 16 mm) without apical LGE, one case with a myocardial phenotype suggestive of Fabry disease characterized by concentric left ventricular hypertrophy, basal inferolateral LGE, and low native T1 values, and one case demonstrating sequelae of prior subclinical myocarditis with extensive subepicardial LGE involving the lateral wall. Two additional athletes fulfilled imaging criteria for structural myocardial disease without a specific syndromic diagnosis.

In three athletes (7.5%), CMR revealed minor myocardial tissue abnormalities of uncertain clinical significance. These findings were defined according to current cardiovascular magnetic resonance recommendations and included small, focal LGE areas involving less than 3% of left ventricular mass, or isolated borderline elevations in native T1 values without associated hypertrophy, ventricular dysfunction, or typical LGE patterns. None of these athletes met established CMR diagnostic criteria for cardiomyopathy or inflammatory myocardial disease.

The distribution of CMR-based diagnoses and their stratification according to referral indication are presented in Table 3.

**Table 3:** Cardiac magnetic resonance (CMR) findings and final diagnostic reclassification according to referral indication Note (important): “non-specific cardiomyopathy phenotype” refers to athletes with objective CMR abnormalities consistent with non-physiological myocardial disease (e.g., non-ischemic LGE pattern and/or focal regional wall motion abnormality and/or mild systolic impairment) but without a definitive syndromic diagnosis (complete description provided in Results). ECG: electrocardiography; LGE: late gadolinium enhancement

Final CMR-based diagnosis	Total (n = 40)	Equivocal echocardiography (n = 25)	Isolated ECG abnormalities (n = 15)
Physiological athletic remodeling	32 (80%)	20	12
Structural cardiomyopathy diagnosed	5 (12.5%)	4	1
– Apical hypertrophic cardiomyopathy	1	1	0
– Fabry disease-like phenotype	1	1	0
– Sequelae of prior myocarditis	1	0	1
– Non-specific cardiomyopathy phenotype	2	2	0
Minor myocardial tissue abnormalities of uncertain significance	3 (7.5%)	1	2
Total	40 (100%)	25 (100%)	15 (100%)

Diagnostic yield of CMR according to referral indication

Among the 25 athletes referred for CMR due to equivocal echocardiographic findings, CMR primarily served a role of diagnostic clarification and reassurance. Physiological cardiac remodeling consistent with athletic adaptation was confirmed in 20 athletes (80%), while structural cardiomyopathy was identified in four cases (16%). Minor myocardial tissue abnormalities of uncertain clinical significance were detected in one athlete (4%).

Among the 15 athletes referred for isolated ECG abnormalities despite normal TTE, CMR confirmed the absence of structural heart disease in 12 athletes (80%). However, clinically relevant myocardial abnormalities or minor tissue abnormalities were identified in three athletes (20%), highlighting the potential added diagnostic value of second-line tissue characterization in this subgroup.

Overall, CMR provided incremental diagnostic value beyond first-line screening in a substantial proportion of referred athletes by enabling reliable differentiation between physiological athletic adaptation and pathological myocardial conditions. Quantitative CMR parameters characterizing physiological athletic remodeling, including increased biventricular volumes, myocardial mass, and preserved systolic function, are summarized in Table 4.

**Table 4:** Quantitative CMR parameters: physiological remodeling group (summary) This table summarizes quantitative CMR measurements of LV and RV volumes, systolic function, myocardial mass, wall thickness, and myocardial tissue characterization in athletes in whom CMR findings were consistent with physiological athletic remodeling. Sedentary reference ranges are provided for contextual comparison and should not be interpreted as pathological thresholds in trained athletes. Data are presented as median (interquartile range). CMR: cardiac magnetic resonance; LGE: late gadolinium enhancement; LV: left ventricle; RV: right ventricle

CMR parameter	Physiological remodeling group (n = 32)	Normal reference ranges (sedentary)
LV end-diastolic volume index (mL/m²)	128 (118–138)	62–96
LV end-systolic volume index (mL/m²)	52 (47–58)	19–39
LV ejection fraction (%)	59 (56–62)	54–74
LV myocardial mass index (g/m²)	92 (85–101)	50–72
Maximal wall thickness (mm)	12 (11–13)	≤12
RV end-diastolic volume index (mL/m²)	122 (112–132)	62–96
RV ejection fraction (%)	55 (52–58)	54–74
Presence of LGE, n (%)	0 (0%)	Absent

In contrast, detailed individual CMR measurements and tissue characteristics of athletes diagnosed with structural cardiomyopathy are presented in Table 5, illustrating the heterogeneity of pathological phenotypes identified within this cohort.

**Table 5:** Quantitative CMR parameters: structural cardiomyopathy cases (individual values) This table summarizes individual CMR measurements, including ventricular volumes, systolic function, myocardial mass, WT, and tissue characterization features, for athletes in whom CMR identified a structural cardiomyopathy. CMR: cardiac magnetic resonance; ECV: extracellular volume fraction; LGE: late gadolinium enhancement; LV: left ventricle; LVEDVI: left ventricular end-diastolic volume index; LVEF: left ventricular ejection fraction; LVESVI: left ventricular end-systolic volume index; RV: right ventricle; RVEDVI: right ventricular end-diastolic volume index; RVEF: right ventricular ejection fraction; T1: native T1 relaxation time; WT: wall thickness

Case	Final diagnosis	LVEDVI (mL/m²)	LVESVI (mL/m²)	LVEF (%)	LV mass index (g/m²)	Max WT (mm)	RVEDVI (mL/m²)	RVEF (%)	LGE present	Key tissue feature
1	Apical hypertrophic cardiomyopathy	78	30	62	115	16	98	58	No	Apical hypertrophy with cavity obliteration and no apical LGE
2	Fabry-like phenotype	85	34	60	108	15	102	56	Yes	Basal inferolateral LGE; low native T1 (610 ms)
3	Prior myocarditis sequelae	102	44	57	75	11	110	54	Yes	Subepicardial LGE along lateral wall (~10% circumference)
4	Non-specific cardiomyopathy phenotype	95	42	56	82	12	105	55	Yes	Mid-wall septal LGE; borderline low-normal LVEF
5	Non-specific cardiomyopathy phenotype	88	36	59	78	11	100	57	No	Borderline high native T1 (1,080 ms) and mildly increased ECV (28%)

## Discussion

In this descriptive study of asymptomatic professional football players referred for CMR following inconclusive first-line cardiovascular screening, several clinically relevant observations emerge. Our findings illustrate the practical diagnostic contribution of CMR in clarifying equivocal echocardiographic findings, identifying previously unrecognized structural myocardial disease, and refining evaluation in athletes with isolated ECG abnormalities. Collectively, these results support the role of CMR as a clinically meaningful second-line imaging modality within contemporary athlete screening pathways, consistent with current international guideline recommendations for selective use [3,10]. Overall, clinically relevant CMR findings were identified in 12.5% of referred athletes, while CMR provided diagnostic reassurance in approximately 80% of cases with equivocal first-line findings.

Differentiating physiological athletic remodeling from pathology

Distinguishing physiological cardiac adaptation from early or atypical cardiomyopathy remains a central challenge in preventive sports cardiology. In highly trained athletes, balanced ventricular enlargement and increased myocardial mass may overlap with pathological phenotypes, particularly hypertrophic and arrhythmogenic cardiomyopathies [11,12]. In our cohort, CMR confirmed physiological athletic remodeling in the majority of referred athletes, allowing confident exclusion of suspected pathology in approximately 80% of cases with equivocal echocardiographic findings.

Quantitative CMR parameters in this group demonstrated increased biventricular volumes and myocardial mass exceeding sedentary reference ranges, preserved systolic function, and the absence of myocardial fibrosis or abnormal tissue characterization. Sedentary reference ranges are provided for contextual comparison rather than diagnostic thresholds in trained athletes. These findings are consistent with previously reported CMR values in endurance-trained and mixed-sport athletes and highlight the intrinsic limitations of TTE when evaluating borderline morphologic measurements in highly trained individuals [11-13].

Importantly, CMR enabled confident exclusion of arrhythmogenic cardiomyopathy in athletes with concerning right ventricular echocardiographic findings. None of the athletes fulfilled current CMR diagnostic criteria for arrhythmogenic cardiomyopathy, underscoring the utility of CMR for comprehensive right ventricular assessment, including accurate volumetric quantification and tissue characterization, which remain challenging to assess reliably with ultrasound alone [12,14].

Detection of clinically silent structural cardiomyopathy

Beyond diagnostic reassurance, CMR identified clinically relevant myocardial disease in a subset of referred athletes. Structural cardiomyopathy or myocardial disease was identified in 12.5% of cases, including apical hypertrophic cardiomyopathy, a Fabry disease-like myocardial phenotype based on characteristic CMR features, without genetic or enzymatic confirmation at the time of screening, and sequelae of prior subclinical myocarditis. These diagnoses were not established during initial echocardiographic screening, highlighting the incremental diagnostic contribution of CMR, particularly through LGE and parametric mapping techniques [15-17].

Apical hypertrophic cardiomyopathy represents a recognized diagnostic challenge on echocardiography, especially in athletes with deep apical cavities or suboptimal acoustic windows. Similarly, infiltrative myocardial diseases such as Fabry disease may present with mild hypertrophy and subtle functional changes, while myocardial fibrosis often represents the earliest detectable abnormality. In these scenarios, CMR offers superior spatial resolution, precise wall thickness assessment, and unique tissue-level information that supports accurate phenotyping and informed clinical management, including follow-up and family evaluation when appropriate [15,18].

CMR in athletes with isolated ECG abnormalities

In athletes referred solely for isolated ECG abnormalities despite normal echocardiographic evaluation, CMR identified clinically relevant myocardial abnormalities or minor tissue alterations in approximately one-fifth of cases. This observation aligns with previous evidence demonstrating that a normal echocardiogram does not fully exclude underlying myocardial disease in athletes with abnormal ECG patterns [16,19]. These findings support current guideline recommendations advocating targeted second-line use of CMR in athletes with unexplained or discordant electrical findings as part of a stepwise screening strategy [3,10].

Minor tissue abnormalities and the persistent diagnostic gray zone

A small subset of athletes demonstrated minor myocardial tissue abnormalities of uncertain clinical significance, such as limited focal LGE or borderline parametric mapping values in the absence of overt cardiomyopathy. These findings reflect a persistent diagnostic gray zone in sports cardiology. Small non-ischemic LGE patterns have been reported in athletes and may represent benign remodeling, prior subclinical injury, or early disease [17,20].

At present, the prognostic significance of such findings remains incompletely defined. In our cohort, these abnormalities did not fulfill established diagnostic criteria for cardiomyopathy or active myocardial inflammation, and no prognostic conclusions can be drawn in the absence of longitudinal outcome data. These observations emphasize the need for cautious interpretation, standardized reporting, and prospective follow-up studies to clarify their clinical relevance [14,20].

Implications for athlete risk stratification, eligibility, and longitudinal follow-up

Beyond diagnosis, the findings of this study have implications for athlete management and follow-up strategies. In competitive sports, the presence of a cardiac abnormality must be interpreted in relation to its association with exercise-related risk. Contemporary sports cardiology emphasizes individualized risk stratification rather than binary diagnostic labeling, and in this context, CMR provides valuable complementary information [21].

In our cohort, athletes in whom CMR confirmed physiological remodeling without myocardial fibrosis were considered at low risk and could be managed conservatively with routine clinical surveillance. In athletes diagnosed with structural cardiomyopathy or myocardial disease, CMR findings supported individualized management strategies incorporating additional investigations, specialist follow-up, and eligibility decisions consistent with contemporary recommendations and shared decision-making principles [22,23].

Furthermore, CMR establishes a robust baseline for longitudinal follow-up. In athletes with confirmed cardiomyopathy, minor tissue abnormalities, or borderline findings, baseline CMR facilitates meaningful comparison over time and supports early detection of disease progression or new tissue abnormalities [24,25].

CMR in a resource-evolving healthcare context

A key strength of this study lies in its real-world context. CMR was integrated as a second-line imaging modality within a selective, indication-driven framework, reflecting the progressive expansion of access to advanced cardiac imaging rather than routine or universal application. This pragmatic approach resulted in a referral rate of 13.3% and a meaningful diagnostic yield.

Our experience suggests that even selective access to CMR can enhance diagnostic accuracy and clinical decision-making in athlete screening. As access to advanced cardiac imaging continues to expand globally, such indication-driven models may help translate technological advances into effective and equitable preventive care [26,27].

Study limitations

Several limitations warrant consideration. The observational, single-center design and selective referral strategy introduce potential referral bias and limit generalizability. The absence of longitudinal follow-up precludes definitive prognostic assessment, particularly for athletes with minor tissue abnormalities. Additionally, the cohort consisted exclusively of male professional football players, and findings may not be directly applicable to female athletes or other sporting disciplines. Accordingly, these findings should be interpreted as a descriptive, hypothesis-generating experience rather than evidence of diagnostic performance or outcome prediction.

Clinical implications

Despite these limitations, this study provides descriptive real-world evidence supporting the selective integration of CMR as a second-line tool in athlete cardiovascular screening. Strategic use of CMR facilitates differentiation between physiological adaptation and pathological myocardial conditions, enables detection of clinically silent cardiomyopathies, and improves characterization of ambiguous findings. As access to advanced cardiac imaging evolves, such indication-driven approaches may contribute to optimized athlete care and prevention strategies.

## Conclusions

This real-world experience from a Moroccan tertiary center suggests that CMR can represent a valuable second-line imaging modality in the cardiovascular evaluation of elite football players with inconclusive initial screening. In this cohort, CMR enabled reliable differentiation between physiological athletic remodeling and pathological myocardial disease, providing diagnostic clarification in most cases while identifying clinically silent cardiomyopathies in a subset of athletes.

Beyond diagnostic refinement, CMR provided additional information relevant to individualized risk stratification through myocardial tissue characterization and served as a baseline for longitudinal follow-up. As access to advanced cardiac imaging continues to expand in resource-evolving healthcare systems, a pragmatic, indication-driven integration of CMR into athlete screening pathways may help optimize diagnostic accuracy, support informed eligibility decisions, and contribute to contemporary strategies aimed at reducing the risk of sudden cardiac death in sport.
